# Gender moderates the relationship between childhood abuse and internalizing and substance use disorders later in life: a cross-sectional analysis

**DOI:** 10.1186/s12888-016-1071-7

**Published:** 2016-11-15

**Authors:** Xiangfei Meng, Carl D’Arcy

**Affiliations:** 1 Department of Psychiatry, McGill University and Douglas Mental Health University Institute, 6875 boul. LaSalle, Verdun, Montreal, QC Canada; 2 Department of Psychiatry & School of Public Health, University of Saskatchewan, Saskatoon, SK Canada

**Keywords:** Childhood maltreatment, Internalizing, Substance use, Interaction, Gender

## Abstract

**Background:**

Although some studies examined the moderating role of gender in the relationship between childhood maltreatment and mental disorders later in life, a number of them examined the effects of only one or two types of maltreatment on an individual mental disorder, for instance, depression, substance use. It is of considerable clinical and theoretical importance to have in-depth understanding what roles of different types of childhood abuse play out in a wide range of mental disorders among women and men using well accepted instruments measuring abuse and mental disorders. The present study aimed to examine this issue using a large nationally representative population sample to explore the gender effect of different types of childhood abuse in mental disorders, and assess the moderating role of gender in the abuse-mental disorder relationship.

**Methods:**

Using data from the Canadian Community Health Survey 2012: Mental Health we sought to answer this question. Respondents with information on childhood maltreatment prior to age 16 were selected (*N* = 23, 395).

**Results:**

We found: i) strong associations between childhood abuse frequency and gender; ii) significant differences between men and women in terms of mental disorders; iii) strong associations between childhood abuse and mental disorders; and, iv) gender moderated the role of childhood abuse history on adulthood mental disorders. Females with a history of sexual abuse and/or exposure to interpersonal violence were at a greater risk of alcohol abuse or dependence later in life.

**Conclusions:**

Intervention should occur as early as possible, and should help female victims of childhood sexual abuse and/or exposure to interpersonal violence, and their families to build more constructive ways to effectively reduce the negative affects of these experiences. Recognition of the moderating role of gender on the relationship between childhood abuse history and mental disorders later in life may aid clinicians and researchers in providing optimal health services.

## Background

Childhood maltreatment, or abuse, is a major public health and social welfare problem. It includes psychological abuse, physical abuse, sexual abuse and neglect [1]. It can cause death, disability, and long-term consequences that result in actual or potential harm to a child’s health, development into adulthood and their dignity. A WHO 2006 report on children maltreatment stressed the need to draw public attention to its health and behavioural impact and that investment in resources is required to both monitor and prevent its occurrence [2].

It is estimated that every year worldwide about 4–16 % of children are physically abused, 10 % are neglected or psychologically abused, 5 to 10 % of girls and up to 5 % of boys are subject to penetrative sexual abuse [3]. Childhood maltreatment significantly contributes to child mortality, morbidity, and other lasting effects on physical and mental health, school performance, alcohol and illegal drug misuse, and criminal behaviour [4].

Studies on childhood maltreatment have identified that: childhood maltreatment is prevalent and has a significant consequence on psychological and health outcomes [1, 3, 5]; several parental and child factors are associated with high risk of being maltreated [6, 7]; boys are more likely to have experienced physical abuse, but girls tend to have more negative consequences of physical abuse, and were more likely to be sexually abused [8, 9]; and, effective prevention programs are recommended to prevent the occurrence of maltreatment [10, 11]. Adverse childhood experiences are associated with enduring changes to the child’s nervous, endocrine and immune systems [12].

Epidemiological studies have consistently found the gender differences in many mental disorders, for example, women are more likely to suffer mood and anxiety disorders [13, 14], but they report lower rates of substance abuse than men [15]. Large-scale epidemiological studies have consistent identified gender differences in the prevalence of internalizing and substance use disorders in the general population, with females suffering an increased risk of internalizing disorders and males of externalizing disorders [16, 17]. A cross-national study of countries in Africa, the Americas, Europe, the Middle East, and the Pacific also found women had more internalizing disorders, whereas men had more externalizing disorders – alcohol and drug substance use [18]. Recently, Steel et al. [19] in their systematic review synthesized the evidence over 174 surveys across 63 countries found that women were more likely to experience mood and anxiety disorders, whereas men were more likely to have substance use disorders.

Although some studies examined the moderating role of gender in the relationship between childhood maltreatment and adult mental disorders, a number of them examined only one or two types of maltreatment on an individual mental disorder, for instance, depression, substance use, etc. [20, 21]. Findings on the moderating role of gender in the abuse-mental disorder relationship are inconsistent across studies. One explanation of this inconsistency is the application of different psychometric properties of abuse measures and diagnostic instruments of mental disorders [21]. Furthermore, Kristman-Valente & Well [22] in their systematic review of longitudinal studies analyzed the role of gender in the association between childhood maltreatment and substance use and because of differences in measurement, sample composition, and developmental timing of substance use, concluded that the moderating effect of gender remained unclear, as very few studies had been conducted to explore the role of gender in this relationship.

It is of considerable clinical and theoretical importance to have in-depth understanding what roles of different types of childhood abuse play out in a wide range of mental disorders among women and men using well accepted instruments measuring abuse and mental disorders. The present study aimed to meet this need by using a large nationally representative population sample to explore the gender effect of childhood abuse in mental disorders, and assess the moderating role of gender in the childhood abuse-mental disorder relationship.

## Methods

### Data source

Data was from the master file of the Canadian Community Health Survey 2012: Mental Health (CCHS 2012-MH). Permission was granted for analyzing this master file of the CCHS 2012-MH from Statistics Canada. This survey is a cross-sectional study that collected information about lifetime and 12-month mental health status, access to and perceived need for formal and informal services and supports, functioning and disability, and factors determining health status. The survey used a multistage stratified cluster sampling design to recruit a representative sample of respondents aged 15 and over residing in the 10 provinces of Canada (not including those residents living in the three territories, indigenous communities, and institutions, and full-time members of the Canadian Armed Forces). The overall response rate was 79.8 %, resulting in a total sample size of 25,113 respondents. The Canadian Community Health Survey 2012: Mental Health was approved by the Statistics Canada ethics review committee. It was an interview survey using computer assisted personal interviewing technology. Respondents were informed and gave written consent to being surveyed. Only respondents aged 18 years and over were asked to answer questions about childhood abuse that occurred before the age of 16 years (*N* = 23,395) and thus included in this study.

### Dependent variables- mental disorders

The survey used the structured Composite International Diagnostic Interview, which is based on the criteria of the Diagnostic and Statistical Manual of Mental disorders, 4^th^ edition, to assess the lifetime and 12-month diagnoses of mental disorders. Disorders assessed included major depression, hypomania, mania, bipolar disorder, generalized anxiety disorder, alcohol abuse or dependence, and drug abuse or dependence. Respondents with major depression, bipolar disorder, generalized anxiety disorder, hypomania, mania, or mood disorder, were further classified as having internalizing disorders. Those with alcohol abuse or dependence or drug abuse or dependence were classified as having externalizing disorders.

### Independent variables

Childhood maltreatment, including physical abuse, sexual abuse, and exposure to interpersonal violence (‘parent hit other adult’), was assessed in the survey using the questions from an abbreviated version of the Childhood Experiences of Violence Questionnaire [23]. To facilitate the respondent’s comfort and privacy, the administration of this module relied on a companion response booklet, which showed both the question and the response categories, so that neither needed to be read aloud. The five categories representing frequency of childhood abuse occurrence were: never, 1–2 times, 3–5 times, 6–10 times, or >10 times.

### Covariates

Socio-demographic variables including age, gender, education, income, marital status, place of residence, immigrant status were considered as covariates in the analyses.

### Statistical analyses

The bootstrapping method recommended by Statistics Canada was used to account for the complex sampling design and make sure the results could be generalized to the general population in Canada. We calculated the overall and gender-specific proportions of all different types of childhood abuse and the gender-specific prevalence for selected mental disorders. The prevalence of selected mental disorders by childhood abuse was estimated. We also determined the distribution of socio-demographic variables by different types of childhood abuse experience and tested the interaction between gender and different types of childhood abuse for selected mental disorders using multivariate logistic regression analyses.

## Results

### Childhood abuse frequency by gender

Table 1 shows that there were significantly statistical differences between men and women for all different types of childhood abuse frequencies (*p* < 0.001), including physical abuse, sexual abuse, exposure to interpersonal violence, and any abuse. Men were more likely exposed to frequent physical abuse, interpersonal violence, and any abuse, whereas women reported more frequent sexual abuse.

**Table 1:** Proportions of childhood abuse frequency by gender

Variable	Men		Women		*P*-value	Total
	%	95 % CI^a^	%	95 % CI		
Physical abuse					<0.001	
Never	26.48	26.02–27.54	32.46	31.72–33.21		
1–2 times	10.29	9.72–10.89	9.67	9.07–10.31		
3–5 times	7.30	6.78–7.86	4.50	4.13–4.90		
6–10 times	2.99	2.69–3.31	2.14	1.89–2.42		
> 10 times	1.93	1.64–2.27	1.94	1.64–2.30		
All						100.0 %
Sexual abuse					*<0.001*	
Never	45.94	45.56–46.33	43.25	42.77–43.72		
1–2 times	1.89	1.64–2.17	4.34	3.99–4.71		
3–5 times	0.42	0.31–0.56	1.39	1.15–1.67		
6–10 times	0.34	0.23–0.51	1.11	0.93–1.34		
> 10 times	0.69	0.51–0.94	0.63	0.47–0.85		
All						100.0 %
Exposure to interpersonal violence					<0.001	
Never	42.04	41.40–42.68	42.89	42.32–43.45		
1–2 times	3.70	3.29–4.15	3.46	3.10–3.86		
3–5 times	1.09	0.91–1.30	1.46	1.23–1.74		
6–10 times	0.49	0.37–0.64	0.57	0.45–0.72		
> 10 times	1.79	1.51–2.13	2.52	2.16–2.94		
All						100.0 %
Any abuse					<0.001	
Never	25.30	24.53–26.08	29.25	28.52–29.99		
1–2 times	10.19	9.64–10.77	9.52	8.95–10.12		
3–5 times	7.47	6.95–8.03	5.50	5.07–5.96		
6–10 times	3.66	3.33–4.02	3.12	2.77–3.50		
> 10 times	2.67	3.33–3.06	3.33	2.96–3.75		
All						100.0 %

^a^95 % CI = 95 % confidence interval

### Socio-demographic variables by different types of childhood abuse

Table 2 reports the distribution of socio-demographic variables among respondents with and without a history of childhood abuse. Respondents aged 45 to 64 years had the highest rates of all kinds of childhood abuse compared to those in other age groups. Females were more likely to report sexual abuse and exposure to interpersonal violence, whereas males more likely to report physical and any abuse (*P* < 0.001). Child abuse was less likely among respondents who were divorced, separated, and widowed than among respondents who were married or in common-law relationship. Those with low- and middle- income were more likely to have a history of abuse in their childhood than people with higher-incomes ($ 50,000 and more). Most respondents reporting childhood abuse had completed post secondary education (*P* < 0.05). Immigrants were less likely to report a history of sexual abuse (*P* < 0.001).

**Table 2:** Socio-demographic profile by types of childhood abuse

Selected characteristics	Physical abuse (%)			Sexual abuse (%)			Exposure to interpersonal violence (%)			Any abuse (%)		
	No	Yes	*P*	No	Yes	*P*	No	Yes	*P*	No	Yes	*P*
Age			<0.001			<0.001			<0.001			<0.001
18–24	13.57	8.56		12.26	4.92		11.88	9.38		13.94	8.67	
25–44	33.15	36.25		35.00	30.40		33.76	39.30		32.97	36.13	
45–64	33.02	39.96		34.75	45.71		35.39	39.41		32.67	39.62	
65+	20.26	15.23		17.99	18.97		18.97	11.92		20.42	15.58	
Gender			<0.001			<0.001			0.100			<0.001
Male	45.20	55.21		51.51	30.90		49.50	46.87		46.37	52.78	
Female	54.80	44.79		48.49	69.10		50.50	53.13		53.63	47.22	
Marital Status			<0.001			<0.001			0.418			<0.001
Married/Common law	56.76	64.92		59.71	63.22		63.28	62.46		55.99	65.00	
Single	30.64	21.69		27.95	19.11		23.38	22.85		31.65	21.41	
Divorced/Separated/Widowed	12.59	13.38		12.34	17.68		13.34	14.70		12.36	13.58	
Income			<0.001			<0.001			<0.001			<0.001
< 15,000	25.97	17.99		22.38	24.09		19.05	23.51		26.40	18.32	
15,000–29,999	21.53	20.49		20.44	26.21		21.37	23.87		20.87	21.32	
30,000–49,999	23.12	24.03		23.58	22.95		24.72	22.02		23.21	23.84	
50,000–69,999	13.66	16.12		14.85	13.53		15.58	14.10		13.76	15.75	
70,000–89,999	7.39	9.58		8.58	6.29		8.66	8.75		7.21	9.56	
90,000+	8.34	11.79		10.17	6.94		10.62	7.76		8.55	11.21	
Education			<0.001			0.039			<0.001			<0.001
Less than secondary	21.84	12.72		18.21	17.35		14.17	16.89		22.28	13.13	
Secondary graduation	16.39	14.87		16.06	13.36		16.37	16.83		16.45	14.95	
Some post secondary	6.80	7.58		6.96	8.40		6.45	8.97		6.53	7.81	
Post secondary	54.97	64.83		58.77	60.90		63.01	57.30		54.73	64.11	
Place of residence			0.181			0.236			0.143			0.404
Urban	81.94	83.24		82.63	81.15		82.19	84.00		82.12	82.90	
Rural	18.06	16.76		17.37	18.85		17.81	16.00		17.88	17.10	
Immigrant			0.218			<0.001			0.161			0.054
Yes	25.67	24.39		25.76	20.01		25.45	27.47		26.05	24.07	
No	74.33	75.61		74.24	79.99		74.55	72.53		73.95	75.93	

### Prevalence of selected mental disorders by gender

The lifetime prevalence of internalizing disorders was 12.75 % in men and 20.47 % in women. In contrast, the lifetime prevalence of substance use disorders was 30.36 % in men and only 12.24 % in women. The past 12-month prevalence of internalizing disorders was 5.24 % among men and 7.71 % among women. The past 12-month prevalence of substance use disorders was 6.19 % among men and 2.42 % among women. Table 3 presents prevalence rates for individual mental disorder by gender.

**Table 3:** Prevalence of selected mental disorders by gender

Selected mental disorders	Men		Women		*P*-value
	%	95 % CI^a^	%	95 % CI	
Internalizing disorders *lifetime*					
Bipolar disorder	2.71	2.33–3.16	2.53	2.11–3.03	0.559
Depression	8.49	7.73–9.32	14.05	13.10–15.06	<0.001
Generalized anxiety disorder	6.04	5.46–6.68	11.32	10.42–12.28	<0.001
Hypomania	1.81	1.50–2.18	1.69	1.35–2.12	0.658
Mania	2.71	2.32–3.15	2.53	2.11–3.03	0.574
Mood disorder	10.03	9.20–10.93	15.06	14.09–16.10	<0.001
Any kind of internalizing disorders	12.75	11.85–13.70	20.47	19.31–21.69	<0.001
Internalizing disorders *12-month*					
Bipolar disorder	1.58	1.28–1.96	1.44	1.13–1.82	0.551
Depression	3.62	3.17–4.14	5.79	5.15–6.52	<0.001
Generalized anxiety disorder	1.95	1.62–2.35	3.17	2.75–3.64	<0.001
Hypomania	0.98	0.74–1.30	0.74	0.55–0.99	0.176
Mania	0.91	0.68–1.21	1.11	0.85–1.45	0.312
Mood disorder	4.37	3.86–4.95	6.41	5.74–7.16	<0.001
Any kind of internalizing disorders	5.24	4.67–5.87	7.71	7.00–8.49	<0.001
Externalizing disorders *lifetime*					
Alcohol abuse or dependence	26.60	25.28–27.97	9.83	9.08–10.64	<0.001
Drug abuse or dependence	11.64	10.70–12.66	5.56	4.99–6.20	<0.001
Any kind of externalizing disorders	30.36	28.96–31.80	12.24	11.37–13.16	<0.001
Externalizing disorders *12-month*					
Alcohol abuse or dependence	4.67	4.09–5.34	1.71	1.42–2.04	<0.001
Drug abuse or dependence	2.51	2.15–2.93	1.07	0.86–1.34	<0.001
Any kind of externalizing disorders	6.19	5.55–6.91	2.42	2.08–2.82	<0.001

^a^95 % CI = 95 % confidence interval

### Prevalence of selected mental disorders by types of childhood abuse

Childhood abuse was consistently associated with the increased risk of the studied mental disorders (*P* < 0.001). There were significant associations between different types of childhood abuse and individual mental disorders. Table 4 shows the detailed relations between types of childhood abuse and mental disorders. Some of 23.62 % of people with any abuse in the childhood were determined to have had an internalizing problem during their lifetime. There were 30.62 % of people with any abuse who had a lifetime externalizing disorder.

**Table 4:** Prevalence of selected mental disorders by types of childhood abuse^a^

Selected mental disorders	Physical abuse	Sexual abuse	Exposure to parent hit other adult	Any abuse
	% (95 % CI)^b^	% (95 % CI)	% (95 % CI)	% (95 % CI)
Internalizing disorders *lifetime*				
Bipolar disorder	3.90 (3.33–4.56)	5.82 (4.77–7.09)	6.55 (5.24–8.16)	3.97 (3.43–4.59)
Depression	16.12 (14.98–17.34)	11.31 (10.69–11.96)	19.01 (16.99–21.21)	16.42 (15.32–17.58)
Generalized anxiety disorder	12.72 (11.77–13.73)	19.91 (17.76–22.25)	15.58 (13.84–17.50)	12.79 (11.89–13.75)
Hypomania	2.48 (2.03–3.03)	3.51 (2.74–4.48)	4.30 (3.20–5.76)	2.51 (2.08–3.03)
Mania	3.88 (3.31–4.54)	5.95 (4.89–7.23)	6.48 (5.18–8.09)	3.95 (3.41–4.57)
Mood disorder	17.97 (16.80–19.20)	27.89 (25.27–30.67)	22.20 (20.05–24.52)	18.32 (17.19–19.50)
Any kind of internalizing disorders	23.25 (21.97–24.58)	35.17 (32.29–38.16)	28.59 (26.24–31.07)	23.62 (22.39–24.89)
Internalizing disorders *12-month*				
Bipolar disorder	2.45 (2.02–2.98)	3.55 (2.72–4.63)	3.59 (2.77–4.64)	2.38 (1.98–2.85)
Depression	7.08 (6.31–7.93)	11.40 (9.55–13.55)	9.01 (7.64–10.59)	6.95 (6.25–7.71)
Generalized anxiety disorder	3.87 (3.38–4.42)	6.23 (5.25–7.38)	5.28 (4.33–6.44)	3.83 (3.37–4.35)
Hypomania	1.32 (1.03–1.69)	1.68 (1.24–2.28)	2.33 (1.71–3.17)	1.29 (1.03–1.63)
Mania	1.65 (1.30–2.10)	2.43 (1.88–3.12)	2.33 (1.77–3.05)	1.60 (1.27–2.00)
Mood disorder	8.19 (7.39–9.06)	12.98 (11.08–15.14)	10.69 (9.26–12.32)	8.03 (7.31–8.81)
Any kind of internalizing disorders	9.66 (8.81–10.57)	14.83 (12.88–17.03)	12.62 (11.10–14.31)	9.44 (8.68–10.26)
Externalizing disorders *lifetime*				
Alcohol abuse or dependence	27.26 (25.82–28.75)	24.61 (22.33–27.04)	27.57 (25.40–29.84)	26.19 (24.86–27.57)
Drug abuse or dependence	14.21 (13.02–15.49)	16.06 (13.95–18.42)	16.48 (14.43–18.75)	13.56 (12.47–14.72)
Any kind of externalizing disorder	31.77 (30.20–33.38)	29.69 (27.15–32.36)	33.00 (30.50–35.60)	30.62 (29.15–32.13)
Externalizing disorders *12-month*				
Alcohol abuse or dependence	4.40 (3.82–5.08)	4.08 (2.99–5.54)	4.71 (3.72–5.96)	4.10 (3.57–4.72)
Drug abuse or dependence	2.50 (2.11–2.97)	2.73 (2.02–3.68)	3.06 (2.37–3.94)	2.44 (2.07–2.86)
Any kind of externalizing disorder	5.87 (5.21–6.62)	5.31 (4.14–6.78)	6.67 (5.54–8.01)	5.57 (4.96–6.24)

^a^ *P* < 0.001

^b^95 % CI = 95 % confidence interval

### Relationships between mental disorders and childhood abuse moderated by gender

Interactions between gender and different types of child abuse were tested for each mental disorder without and with adjusting for socio-demographic variables. Table 5 summarizes the significant interactions between gender and type of child abuse for selected mental disorders. Significant interactions were found between being female and sexual abuse for alcohol abuse or dependence (adjusted OR for the interaction: 1.46, 95 % CI 1.04-2.04) and between being female and exposure to interpersonal violence for alcohol abuse or dependence (adjusted OR for the interaction: 1.41, 95 % CI 1.06–1.86). Though, females were less likely to have alcohol abuse or dependence or substance use disorders, after adjusting other socio-demographic variables, the statistical interactions between sexual abuse and exposure to interpersonal violence and being female indicate persons with these combinations of attributes were more likely to report suffering from alcohol abuse or dependence. Tables 6, 7, 8, and 9 in Appendix provides details on the interactions being female, types of child abuse and specific psychiatric diagnoses.

**Table 5:** Significant relationships between studied mental disorders and childhood abuse moderated by female gender

Childhood abuse and mental disorders	Type of abuse		Female gender		Interactions^b^	
	OR (95 % CI)	*P*-value	OR (95 % CI)	*P*-value	OR (95 % CI)	*P*-value
Physical abuse						
Alcohol abuse or dependence past 12-month	1.56 (1.16–2.09)	0.003	0.29 (0.21–0.41)	<0.001	1.57 (1.00–2.48)	0.05
Alcohol abuse or dependence past 12-month^a^	1.97 (1.37–2.85)	<0.001	0.29 (0.19–0.43)	<0.001	1.51 (0.87–2.59)	0.13
Sexual abuse						
Alcohol abuse or dependence lifetime	1.66 (1.30–2.12)	<0.001	0.26 (0.23–0.29)	<0.001	1.60 (1.17–2.19)	0.003
Alcohol abuse or dependence lifetime^a^	1.66 (1.27–2.17)	<0.001	0.25 (0.22–0.29)	<0.001	1.46 (1.04–2.04)	0.03
Externalizing problems lifetime	1.89 (1.46–2.44)	<0.001	0.28 (0.25–0.31)	<0.001	1.39 (1.02–1.90)	0.04
Externalizing problems lifetime^a^	2.09 (1.59–2.75)	<0.001	0.26 (0.23–0.29)	<0.001	1.19 (0.85–1.67)	0.31
Exposure to “parent hit other adult”						
Alcohol abuse or dependence lifetime	1.65 (1.40–1.95)	<0.001	0.25 (0.22–0.29)	<0.001	1.57 (1.20–2.06)	0.001
Alcohol abuse or dependence lifetime^a^	1.74 (1.45–2.08)	<0.001	0.26 (0.23–0.29)	<0.001	1.41 (1.06–1.86)	0.02
Externalizing problems lifetime	1.89 (1.58–2.25)	<0.001	0.28 (0.25–0.31)	<0.001	1.36 (1.04–1.76)	0.02
Externalizing problems lifetime^a^	2.03 (1.68–2.44)	<0.001	0.27 (0.24–0.31)	<0.001	1.19 (0.90–1.57)	0.23

^a^adjusted for age, marital status, income, education, place of residence, immigrant status

^b^significant interaction terms for studied mental disorders

## Discussion

### Main findings

What this national population survey shows is: 1) strong associations between childhood abuse frequency and gender, with men reporting frequent physical abuse, exposure to interpersonal violence, and any abuse, women more frequently reported sexual abuse; 2) significant differences between men and women in terms of mental disorders, with men having a higher prevalence of substance use disorders and women a higher prevalence of internalizing disorders; 3) strong associations between childhood abuse and mental disorders; 4) after adjusted for socio-demographic variables there were significant statistical interactions between being female and both sexual abuse and exposure to interpersonal violence for alcohol abuse or dependence. Abuse in childhood has a differential impact on females than males.

Although the measurement of child abuse (including its subtypes) may be varied across studies, the major findings of the present study are consistent with previous literature. Studies have consistently showed that childhood abuse is associated with negative mental health outcomes, for example, internalizing disorders [24, 25], externalizing disorders [26, 27], etc. Epidemiological data have also consistently pointed out that there is gender difference in risk of childhood abuse with women are more likely to report a history of childhood sexual abuse [28–30], whereas men are more likely to report physical abuse [8]. The findings for other types of childhood abuse are mixed [22]. Similarly, gender differences have also been identified in many studies of the prevalence and incidence of mental disorders with men having more externalizing disorders, such as alcohol abuse [18, 31, 32], and women having more internalizing disorders, such as depression and anxiety [33, 34].

Notably, in this large-scale population analysis, we found that even though females had a lower risk of substance use disorders, the moderating role of being female in the relationship between sexual abuse and/or exposure to interpersonal violence and substance use disorders significantly increased the vulnerability of females having substance use disorders. Similarly, the interaction between being female and a history of child abuse significantly increased the likelihood of alcohol problems [35]. Consistent with our findings of significant interactions between being female and childhood abuse, a prospective study of African-American women over 20-year period found that childhood sexual abuse was an important predictor of heavy alcohol use and binge drinking in adulthood [36]. A review on child sexual and physical abuse and alcoholism suggested females were more likely to have alcohol problems when they had been exposed to sexual or physical abuse as children, but there was not sufficient evidence to support a similar conclusion for males [37]. There have been numerous studies into the negative impact of child abuse on psychological well-being [38], self-harm [39], self-esteem [40], life satisfaction [41], etc. The moderating role of female gender in the abuse-substance use relationship could be explained by: i) the use of substances as self-medication in an attempt to control over the negative experience; ii) substance use is a way of exhibiting self-destructive behaviour. Therefore, it is important to anticipate the link between the history of childhood sexual abuse and/or exposure to interpersonal violence and adulthood substance use disorders among female child abuse victims. Factors such as positive or adaptive coping skills may help mediate the relationship between childhood victimization and substance use problems. Implications of this finding is that intervention should occur as early as possible, and should be focused on females, who have been sexually abused or exposed to interpersonal violence, and their families to learn more constructive ways of coping with the negative affects that are associated with these adverse experiences.

### Strengths and limitations

This study has several methodological strengths: 1) data used was from a large population-based sample broadly representative of the Canadian population, therefore findings of this study are more reliable and highly generalizable; 2) this study explored interactions between different types of childhood abuse and gender in several selected mental disorders. However the limitations of the study are: 1) the survey is cross-sectional, therefore no causal inferences between childhood abuse and mental disorders could be made; 2) the survey may have recall bias, as childhood abuse was based on retrospective self-reports; 3) though the survey was designed to explore major mental health problems in Canada only the more common mental disorders were assessed.

## Conclusions

Gender differences both in mental disorders and in different types of child abuse exist in the Canadian population. Gender moderated the role of childhood abuse history on adulthood mental disorders. Females with a history of sexual abuse and/ or exposure to interpersonal violence were at a greater risk of alcohol abuse or dependence. It is important to anticipate the link between the history of childhood sexual abuse and/or exposure to interpersonal violence and adulthood substance use disorders among female victims. Factors such as positive and adaptive coping skills may help mediate the relationship between childhood victimization and substance use problems. Earlier interventions should focus on positive and adaptive coping skills to effectively reduce the onset of substance use problems among female victims. In providing optimal care for those with alcohol abuse disorders it is important to recognize and understand the different mechanisms of action and impact of childhood maltreatment for women and men.

## Appendix

**Table 6:** Multivariate analysis for interactions between physical abuse, being female and selected mental disorders

Selected mental disorders	Physical abuse		Female gender		Interactions	
	OR (95 % CI)	*P*-value	OR (95 % CI)	*P*-value	OR (95 % CI)	*P*-value
Internalizing disorders *lifetime*						
Bipolar disorder	2.11 (1.49–3.00)	<0.001	0.91 (0.62–1.35)	0.65	1.18 (0.71–1.95)	0.52
Bipolar disorder^a^	2.66 (1.77–3.99)	<0.001	0.84 (0.53–1.33)	0.45	0.96 (0.55–1.67)	0.87
Depression	2.29 (1.87–2.79)	<0.001	1.87 (1.54–2.28)	<0.001	1.07 (0.83–1.38)	0.63
Depression^a^	2.52 (2.04–3.13)	<0.001	1.85 (1.48–2.32)	<0.001	0.90 (0.68–1.18)	0.45
Generalized anxiety disorder	2.24 (1.80–2.79)	<0.001	1.99 (1.58–2.50)	<0.001	1.18 (0.89–1.58)	0.25
Generalized anxiety disorder^a^	2.20 (1.74–2.79)	<0.001	1.83 (1.42–2.37)	<0.001	1.09 (0.80–1.48)	0.58
Hypomania	1.84 (1.22–2.76)	0.004	0.90 (0.57–1.42)	0.65	1.21 (0.66–2.22)	0.54
Hypomania^a^	2.42 (1.53–3.83)	<0.001	0.85 (0.49–1.49)	0.58	0.92 (0.47–1.80)	0.81
Mania	2.07 (1.46–2.94)	<0.001	0.91 (0.62–1.34)	0.64	1.19 (0.72–1.97)	0.50
Mania^a^	2.63 (1.75–3.95)	<0.001	0.84 (0.53–1.33)	0.46	0.95 (0.54–1.67)	0.86
Mood disorder	2.30 (1.91–2.77)	<0.001	1.69 (1.41–2.03)	<0.001	1.06 (0.83–1.35)	0.63
Mood disorder^a^	2.62 (2.14–3.21)	<0.001	1.71 (1.38–2.12)	<0.001	0.87 (0.67–1.13)	0.30
Any kind of internalizing disorders	2.22 (1.89–2.60)	<0.001	1.83 (1.55–2.16)	<0.001	1.12 (0.90–1.39)	0.31
Any kind of internalizing disorders^a^	2.40 (2.01–2.85)	<0.001	1.85 (1.53–2.22)	<0.001	0.95 (0.75–1.20)	0.67
Internalizing disorders *12-month*						
Bipolar disorder	2.83 (1.74–4.59)	<0.001	0.97 (0.56–1.69)	0.93	1.05 (0.51–2.14)	0.90
Bipolar disorder^a^	4.00 (2.25–7.11)	<0.001	0.94 (0.47–1.90)	0.87	0.80 (0.35–1.79)	0.58
Depression	2.59 (1.90–3.53)	<0.001	1.85 (1.38–2.48)	<0.001	0.96 (0.65–1.41)	0.83
Depression^a^	3.02 (2.16–4.21)	<0.001	1.49 (1.05–2.12)	0.03	0.88 (0.58–1.33)	0.54
Generalized anxiety disorder	2.12 (1.43–3.13)	<0.001	1.53 (1.03–2.28)	0.03	1.30 (0.80–2.11)	0.30
Generalized anxiety disorder^a^	2.14 (1.37–3.35)	0.001	1.18 (0.75–1.87)	0.47	1.20 (0.69–2.07)	0.51
Hypomania	2.84 (1.54–5.23)	0.001	1.01 (0.52–1.99)	0.97	0.71 (0.30–1.67)	0.43
Hypomania^a^	5.14 (2.67–9.86)	<0.001	1.33 (0.59–3.03)	0.49	0.41 (0.16–1.04)	0.06
Mania	3.40 (1.79–6.46)	<0.001	1.56 (0.75–3.25)	0.24	0.82 (0.33–2.07)	0.68
Mania^a^	6.27 (3.00–13.07)	<0.001	2.14 (0.88–5.19)	0.09	0.44 (0.16–1.19)	0.11
Mood disorder	2.72 (2.05–3.62)	<0.001	1.74 (1.31–2.30)	<0.001	0.92 (0.64–1.32)	0.66
Mood disorder^a^	3.37 (2.78–4.58)	<0.001	1.52 (1.08–2.14)	0.02	0.78 (0.53–1.16)	0.22
Any kind of internalizing disorders	2.41 (1.89–3.07)	<0.001	1.60 (1.25–2.05)	<0.001	1.06 (0.77–1.46)	0.70
Any kind of internalizing disorders^a^	2.88 (2.20–3.77)	<0.001	1.38 (1.04–1..84)	0.03	0.91 (0.64–1.93)	0.58
Externalizing disorders *lifetime*						
Alcohol abuse or dependence	2.54 (2.22–2.89)	<0.001	0.30 (0.26–0.36)	<0.001	1.10 (0.88–1.38)	0.41
Alcohol abuse or dependence^a^	2.44 (2.10–2.84)	<0.001	0.30 (0.25–0.35)	<0.001	1.03 (0.81–1.32)	0.79
Drug abuse or dependence	3.07 (2.56–3.69)	<0.001	0.46 (0.38–0.57)	<0.001	1.10 (0.83–1.45)	0.51
Drug abuse or dependence^a^	3.39 (2.76–4.18)	<0.001	0.42 (0.33–0.53)	<0.001	0.98 (0.71–1.36)	0.91
Any kind of externalizing disorders	2.69 (2.37–3.05)	<0.001	0.33 (0.29–0.38)	<0.001	1.04 (0.85–1.28)	0.68
Any kind of externalizing disorders^a^	2.74 (2.37–3.17)	<0.001	0.31 (0.26–0.37)	<0.001	0.96 (0.76–1.21)	0.72
Externalizing disorders *12-month*						
Alcohol abuse or dependence	1.56 (1.16–2.09)	0.003	0.29 (0.21–0.41)	<0.001	1.57 (1.00–2.48)	0.05
Alcohol abuse or dependence^a^	1.97 (1.37–2.85)	<0.001	0.29 (0.19–0.43)	<0.001	1.51 (0.87–2.59)	0.13
Drug abuse or dependence	1.94 (1.39–2.73)	<0.001	0.50 (0.33–0.74)	0.001	0.82 (0.46–1.47)	0.51
Drug abuse or dependence^a^	2.91 (1.94–4.37)	<0.001	0.36 (0.22–0.59)	<0.001	0.83 (0.43–1.59)	0.57
Any kind of externalizing disorders	1.69 (1.32–2.17)	<0.001	0.38 (0.28–0.49)	<0.001	1.11 (0.76–1.62)	0.59
Any kind of externalizing disorders^a^	2.24 (1.63–3.06)	<0.001	0.33 (0.23–0.45)	<0.001	1.12 (0.71–1.75)	0.63

^a^adjusted for age, marital status, income, education, place of residence, immigrant

**Table 7:** Multivariate analysis for interactions between sexual abuse being female and selected mental disorders

Selected mental disorders	Sexual abuse		Female gender		Interactions	
	OR (95 % CI)	*P*-value	OR (95 % CI)	*P*-value	OR (95 % CI)	*P*-value
Internalizing disorders *lifetime*						
Bipolar disorder	3.20 (2.15–4.76)	<0.001	0.87 (0.64–1.17)	0.35	0.81 (0.46–1.40)	0.45
Bipolar disorder^a^	3.54 (2.24–5.58)	<0.001	0.72 (0.51–1.01)	0.06	0.73 (0.39–1.37)	0.33
Depression	2.31 (1.71–3.11)	<0.001	1.51 (1.31–1.74)	<0.001	1.37 (0.96–1.95)	0.09
Depression^a^	2.43 (1.76–3.37)	<0.001	1.38 (1.18–1.63)	<0.001	1.20 (0.82–1.75)	0.36
Generalized anxiety disorder	2.74 (2.02–3.71)	<0.001	1.78 (1.52–2.09)	<0.001	1.03 (0.73–1.47)	0.85
Generalized anxiety disorder^a^	2.64 (1.87–3.72)	<0.001	1.62 (1.36–1.94)	<0.001	0.94 (0.63–1.40)	0.78
Hypomania	3.33 (2.04–5.46)	<0.001	0.95 (0.66–1.37)	0.80	0.57 (0.30–1.08)	0.09
Hypomania^a^	3.86 (2.28–6.54)	<0.001	0.81 (0.53–1.24)	0.34	0.49 (0.24–1.04)	0.05
Mania	3.30 (2.23–4.90)	<0.001	0.87 (0.64–1.17)	0.35	0.81 (0.47–1.40)	0.44
Mania^a^	3.69 (2.35–5.82)	<0.001	0.72 (0.51–1.02)	0.06	0.72 (0.38–1.34)	0.30
Mood disorder	2.50 (1.90–3.29)	<0.001	1.37 (1.20–1.56)	<0.001	1.30 (0.94–1.80)	0.12
Mood disorder^a^	2.72 (2.00–3.70)	<0.001	1.26 (1.08–1.47)	0.003	1.13 (0.79–1.62)	0.50
Any kind of internalizing disorders	2.43 (1.89–3.13)	<0.001	1.53 (1.36–1.73)	<0.001	1.32 (0.98–1.78)	0.07
Any kind of internalizing disorders^a^	2.58 (1.94–3.42)	<0.001	1.44 (1.26–1.65)	<0.001	1.18 (0.84–1.66)	0.33
Internalizing disorders *12-month*						
Bipolar disorder	3.21 (1.94–5.30)	<0.001	0.82 (0.55–1.22)	0.32	0.90 (0.45–1.81)	0.77
Bipolar disorder^a^	3.32 (1.84–6.01)	<0.001	0.66 (0.41–1.07)	0.09	0.87 (0.40–1.92)	0.74
Depression	2.84 (1.86–4.34)	<0.001	1.44 (1.17–1.78)	0.001	1.05 (0.63–1.73)	0.86
Depression^a^	2.81 (1.77–4.84)	<0.001	1.09 (0.85–1.41)	0.49	1.09 (0.63–1.88)	0.77
Generalized anxiety disorder	3.20 (2.02–5.06)	<0.001	1.52 (1.14–2.01)	0.004	0.84 (0.50–1.43)	0.52
Generalized anxiety disorder^a^	2.81 (1.69–4.67)	<0.001	1.16 (0.85–1.59)	0.35	0.83 (0.46–1.49)	0.53
Hypomania	3.03 (1.65–5.57)	<0.001	0.75 (0.46–1.23)	0.25	0.65 (0.30–1.43)	0.29
Hypomania^a^	3.83 (2.04–7.18)	<0.001	0.66 (0.35–1.25)	0.21	0.56 (0.24–1.30)	0.18
Mania	3.84 (2.06–7.16)	<0.001	1.20 (0.74–1.96)	0.46	0.64 (0.29–1.39)	0.26
Mania^a^	3.76 (1.82–7.74)	<0.001	1.07 (0.60–1.90)	0.81	0.54 (0.22–1.33)	0.18
Mood disorder	2.91 (1.98–4.26)	<0.001	1.32 (1.07–1.62)	0.01	1.04 (0.66–1.65)	0.86
Mood disorder^a^	3.00 (1.95–4.60)	<0.001	1.03 (0.80–1.32)	0.82	1.07 (0.64–1.77)	0.80
Any kind of internalizing disorders	2.85 (1.99–4.08)	<0.001	1.36 (1.13–1.63)	0.001	0.99 (0.65–1.52)	0.96
Any kind of internalizing disorders^a^	2.82 (1.88–4.23)	<0.001	1.05 (0.85–1.31)	0.64	1.04 (0.65–1.66)	0.88
Externalizing disorders *lifetime*						
Alcohol abuse or dependence	1.66 (1.30–2.12)	<0.001	0.26 (0.23–0.29)	<0.001	1.60 (1.17–2.19)	0.003
Alcohol abuse or dependence^a^	1.66 (1.27–2.17)	<0.001	0.25 (0.22–0.29)	<0.001	1.46 (1.04–2.04)	0.03
Drug abuse or dependence	2.60 (1.88–3.59)	<0.001	0.38 (0.32–0.45)	<0.001	1.22 (0.81–1.85)	0.34
Drug abuse or dependence^a^	2.96 (2.05–4.27)	<0.001	0.34 (0.28–0.41)	<0.001	1.01 (0.63–1.62)	0.96
Any kind of externalizing disorders	1.89 (1.46–2.44)	<0.001	0.28 (0.25–0.31)	<0.001	1.39 (1.02–1.90)	0.04
Any kind of externalizing disorders^a^	2.09 (1.59–2.75)	<0.001	0.26 (0.23–0.29)	<0.001	1.19 (0.85–1.67)	0.31
Externalizing disorders *12-month*						
Alcohol abuse or dependence	1.67 (0.96–2.91)	0.07	0.33 (0.26–0.43)	<0.001	1.04 (0.54–2.01)	0.90
Alcohol abuse or dependence^a^	2.09 (1.13–3.87)	0.02	0.33 (0.25–0.45)	<0.001	0.91 (0.44–1.89)	0.80
Drug abuse or dependence	1.99 (1.16–3.42)	0.01	0.39 (0.28–0.54)	<0.001	1.05 (0.51–2.15)	0.90
Drug abuse or dependence^a^	2.76 (1.54–4.91)	0.001	0.27 (0.17–0.41)	<0.001	1.09 (0.51–2.33)	0.83
Any kind of externalizing disorders	1.52 (0.96–2.42)	0.07	0.35 (0.28–0.44)	<0.001	1.13 (0.65–1.96)	0.67
Any kind of externalizing disorders^a^	2.01 (1.19–3.39)	0.01	0.31 (0.24–0.39)	<0.001	1.05 (0.56–1.95)	0.88

^a^adjusted for age, marital status, income, education, place of residence, immigrant

**Table 8:** Multivariate analyses for interactions between interpersonal violence, being female and selected mental disorders

Selected mental disorders	Exposure to parent hit other adult		Female gender		Interactions	
	OR (95 % CI)	*P*-value	OR (95 % CI)	*P*-value	OR (95 % CI)	*P*-value
Internalizing disorders *lifetime*						
Bipolar disorder	3.66 (2.54–5.28)	<0.001	0.88 (0.65–1.20)	0.43	1.08 (0.61–1.90)	0.79
Bipolar disorder^a^	3.50 (2.32–5.29)	<0.001	0.75 (0.54–1.04)	0.09	0.93 (0.52–1.67)	0.80
Depression	1.99 (1.57–2.52)	<0.001	1.70 (1.46–1.98)	<0.001	1.05 (0.76–1.46)	0.75
Depression^a^	2.04 (1.58–2.64)	<0.001	1.59 (1.34–1.87)	<0.001	0.95 (0.67–1.35)	0.79
Generalized anxiety disorder	1.93 (1.52–2.44)	<0.001	1.84 (1.56–2.18)	<0.001	1.19 (0.87–1.61)	0.27
Generalized anxiety disorder^a^	1.80 (1.40–23.32)	<0.001	1.69 (1.41–2.04)	<0.001	1.17 (0.85–1.62)	0.34
Hypomania	3.44 (2.22–5.32)	<0.001	0.88 (0.61–1.26)	0.48	1.12 (0.56–2.24)	0.74
Hypomania^a^	3.49 (2.16–5.64)	<0.001	0.76 (0.51–1.12)	0.17	0.92 (0.46–1.83)	0.81
Mania	3.65 (0.52–5.28)	<0.001	0.89 (0.66–1.22)	0.48	1.06 (0.60–1.88)	0.84
Mania^a^	3.49 (2.31–5.27)	<0.001	0.76 (0.54–1.05)	0.10	0.91 (0.50–1.64)	0.75
Mood disorder	2.36 (1.90–2.94)	<0.001	1.59 (1.38–1.84)	<0.001	0.94 (0.70–1.26)	0.67
Mood disorder^a^	2.44 (1.92–3.10)	<0.001	1.48 (1.26–1.74)	<0.001	0.84 (0.61–1.15)	0.27
Any kind of internalizing disorders	2.23 (1.84–2.70)	<0.001	1.74 (1.53–1.98)	<0.001	1.03 (0.79–1.34)	0.82
Any kind of internalizing disorders^a^	2.26 (1.83–2.78)	<0.001	1.64 (1.42–1.89)	<0.001	0.94 (0.71–1.25)	0.69
Internalizing disorders *12-month*						
Bipolar disorder	3.36 (2.00–5.63)	<0.001	0.85 (0.54–1.34)	0.48	1.08 (0.52–2.24)	0.83
Bipolar disorder^a^	3.27 (1.83–5.82)	<0.001	0.75 (0.47–1.21)	0.24	0.86 (0.40–1.83)	0.70
Depression	2.65 (1.90–3.70)	<0.001	1.59 (1.26–2.01)	<0.001	0.86 (0.55–1.34)	0.50
Depression^a^	2.74 (1.91–3.92)	<0.001	1.35 (1.04–1.75)	0.02	0.72 (0.45–1.17)	0.19
Generalized anxiety disorder	2.31 (1.56–3.42)	<0.001	1.46 (1.10–1.95)	0.10	1.16 (0.73–1.86)	0.53
Generalized anxiety disorder^a^	2.12 (1.37–3.28)	0.001	1.19 (0.88–1.61)	0.25	1.07 (0.64–1.80)	0.80
Hypomania	5.31 (2.79–10.10)	<0.001	0.88 (0.49–1.57)	0.67	0.64 (0.27–1.53)	0.32
Hypomania^a^	6.44 (3.30–12.56)	<0.001	0.90 (0.49–1.66)	0.75	0.43 (0.18–1.04)	0.06
Mania	3.39 (1.77–6.46)	<0.001	1.20 (0.67–2.16)	0.54	0.98 (0.43–2.25)	0.96
Mania^a^	3.31 (1.62–6.73)	0.001	1.14 (0.62–2.09)	0.67	0.72 (0.31–1.71)	0.46
Mood disorder	2.90 (2.14–3.92)	<0.001	1.51 (1.20–1.89)	<0.001	0.83 (0.55–1.24)	0.36
Mood disorder^a^	2.97 (2.13–4.14)	<0.001	1.28 (1.00–1.65)	0.05	0.69 (0.45–1.08)	0.11
Any kind of internalizing disorders	2.74 (2.08–3.61)	<0.001	1.49 (1.23–1.82)	<0.001	0.88 (0.61–1.27)	0.49
Any kind of internalizing disorders^a^	2.69 (1.99–3.65)	<0.001	1.26 (1.01–1.56)	0.04	0.77 (0.52–1.16)	0.21
Externalizing disorders *lifetime*						
Alcohol abuse or dependence	1.65 (1.40–1.95)	<0.001	0.25 (0.22–0.29)	<0.001	1.57 (1.20–2.06)	0.001
Alcohol abuse or dependence^a^	1.74 (1.45–2.08)	<0.001	0.26 (0.23–0.29)	<0.001	1.41 (1.06–1.86)	0.02
Drug abuse or dependence	2.42 (1.90–3.08)	<0.001	0.39 (0.33–0.47)	<0.001	1.24 (0.87–1.77)	0.24
Drug abuse or dependence^a^	2.46 (1.92–3.16)	<0.001	0.37 (0.30–0.45)	<0.001	1.07 (0.73-.156)	0.74
Any kind of externalizing disorders	1.89 (1.58–2.25)	<0.001	0.28 (0.25–0.31)	<0.001	1.36 (1.04–1.76)	0.02
Any kind of externalizing disorders^a^	2.03 (1.68–2.44)	<0.001	0.27 (0.24–0.31)	<0.001	1.19 (0.90–1.57)	0.23
Externalizing disorders *12-month*						
Alcohol abuse or dependence	1.56 (1.08–2.25)	0.02	0.29 (0.22–0.38)	<0.001	1.52 (0.87–2.65)	0.14
Alcohol abuse or dependence^a^	1.67 (1.12–2.49)	0.01	0.32 (0.24–0.42)	<0.001	1.34 (0.74–2.44)	0.34
Drug abuse or dependence	2.22 (1.50–3.30)	<0.001	0.33 (0.22–0.49)	<0.001	1.20 (0.62–2.33)	0.59
Drug abuse or dependence^a^	2.41 (1.55–3.75)	<0.001	0.29 (0.19–0.44)	<0.001	1.06 (0.53–2.14)	0.86
Any kind of internalizing disorders	1.79 (1.33–2.40)	<0.001	0.31 (0.24–0.39)	<0.001	1.30 (0.81–2.08)	2.07
Any kind of internalizing disorders^a^	1.94 (1.38–2.71)	<0.001	0.31 (0.24–0.40)	<0.001	1.18 (0.71–1.96)	0.53

^a^adjusted for age, marital status, income, education, place of residence, immigrant

**Table 9:** Multivariate analysis for interactions between any abuse, being female and selected mental disorders

Selected mental disorders	Any kind of abuse		Female gender		Interactions	
	OR (95 % CI)	*P*-value	OR (95 % CI)	*P*-value	OR (95 % CI)	*P*-value
Internalizing problems *lifetime*						
Bipolar disorder	2.57 (1.79–3.70)	<0.001	0.93 (0.60–1.42)	0.71	1.11 (0.65–1.90)	0.71
Bipolar disorder^a^	3.53 (2.30–5.41)	<0.001	0.85 (0.50–1.46)	0.56	0.91 (0.49–1.68)	0.76
Depression	2.40 (1.97–2.93)	<0.001	1.68 (1.36–2.06)	<0.001	1.22 (0.94–1.58)	0.14
Depression^a^	2.64 (2.12–3.29)	<0.001	1.62 (1.27–2.05)	<0.001	1.07 (0.80–1.43)	0.64
Generalized anxiety disorder	2.56 (2.07–3.18)	<0.001	1.99 (1.57–2.53)	<0.001	1.11 (0.84–1.49)	0.46
Generalized anxiety disorder^a^	2.48 (1.96–3.13)	<0.001	1.83 (1.40–2.39)	<0.001	1.04 (0.77–1.41)	0.79
Hypomania	1.98 (1.30–3.03)	0.002	0.82 (0.50–1.33)	0.42	1.33 (0.71–2.48)	0.37
Hypomania^a^	2.69 (1.66–4.34)	<0.001	0.75 (0.41–1.38)	0.35	1.08 (0.54–2.17)	0.83
Mania	2.52 (1.75–3.62)	<0.001	0.92 (0.60–1.40)	0.69	1.12 (0.65–1.93)	0.67
Mania^a^	3.49 (2.27–5.37)	<0.001	0.85 (0.50–1.45)	0.55	0.91 (0.49–1.70)	0.76
Mood disorder	2.51 (2.09–3.02)	<0.001	1.56 (1.28–1.89)	<0.001	1.16 (0.91–1.48)	0.23
Mood disorder^a^	2.88 (2.35–3.54)	<0.001	1.55 (1.24–1.94)	<0.001	0.99 (0.75–1.30)	0.92
Internalizing problems	2.38 (2.03–2.79)	<0.001	1.70 (1.43–2.02)	<0.001	1.21 (0.97–1.51)	0.09
Internalizing problems^a^	2.58 (2.16–3.09)	<0.001	1.70 (1.39–2.06)	<0.001	1.05 (0.82–1.35)	0.68
Internalizing problems *12-month*						
Bipolar disorder	3.06 (1.80–5.20)	<0.001	0.97 (0.50–1.86)	0.92	1.01 (0.45–2.22)	0.99
Bipolar disorder^a^	4.68 (2.49–8.81)	<0.001	0.95 (0.40–2.23)	0.90	0.77 (0.30–1.96)	0.58
Depression	2.72 (1.99–3.71)	<0.001	1.81 (1.32–2.48)	<0.001	0.95 (0.64–1.40)	0.78
Depression^a^	3.18 (2.26–4.48)	<0.001	1.40 (0.95–2.07)	0.09	0.92 (0.59–1.43)	0.71
Generalized anxiety disorder	2.30 (1.56–3.38)	<0.001	1.49 (0.98–2.27)	0.06	1.27 (0.77–2.07)	0.35
Generalized anxiety disorder^a^	2.46 (1.57–3.83)	<0.001	1.15 (0.71–1.86)	0.57	1.18 (0.68–2.05)	0.55
Hypomania	2.84 (1.46–5.51)	0.002	0.93 (0.43–2.00)	0.85	0.80 (0.32–2.01)	0.63
Hypomania^a^	5.59 (2.80–11.17)	<0.001	1.24 (0.48–3.20)	0.66	0.46 (0.17–1.29)	0.14
Mania	3.73 (1.84–7.58)	<0.001	1.63 (0.69–3.83)	0.27	0.74 (0.27–2.07)	0.57
Mania^a^	8.61 (3.99–18.60)	<0.001	2.70 (0.98–7.46)	0.06	0.33 (0.11–1.01)	0.05
Mood disorder	2.85 (2.13–3.82)	<0.001	1.70 (1.26–2.31)	0.001	0.91 (0.63–1.32)	0.63
Mood disorder^a^	3.58 (2.60–4.94)	<0.001	1.45 (0.99–2.12)	0.06	0.81 (0.53–1.24)	0.34
Internalizing problems	2.49 (1.95–3.20)	<0.001	1.57 (1.20–2.04)	0.001	1.04 (0.76–1.44)	0.79
Internalizing problems^a^	3.03 (2.30–4.00)	<0.001	1.31 (0.96–1.80)	0.09	0.94 (0.65–1.34)	0.72
Externalizing problems *lifetime*						
Alcohol abuse or dependence	2.57 (2.25–2.94)	<0.001	0.28 (0.24–0.33)	<0.001	1.16 (0.92–1.45)	0.20
Alcohol abuse or dependence^a^	2.47 (2.12–2.89)	<0.001	0.27 (0.22–0.33)	<0.001	1.12 (0.87–1.43)	0.37
Drug abuse or dependence	3.15 (2.61–3.80)	<0.001	0.43 (0.35–0.54)	<0.001	1.13 (0.85–1.50)	0.41
Drug abuse or dependence^a^	3.45 (2.78–4.28)	<0.001	0.38 (0.29–0.50)	<0.001	1.06 (0.75–1.48)	0.74
Externalizing disorders	2.74 (2.41–3.12)	<0.001	0.31 (0.27–0.36)	<0.001	1.09 (0.89–1.34)	0.42
Externalizing disorders^a^	2.08 (2.42–3.26)	<0.001	0.29 (0.24–0.34)	<0.001	1.02 (0.81–1.28)	0.87
Externalizing disorders *12-month*						
Alcohol abuse or dependence	1.47 (1.09–1.97)	0.01	0.29 (0.20–0.40)	<0.001	1.52 (0.98–2.38)	0.06
Alcohol abuse or dependence^a^	1.87 (1.29–2.70)	0.001	0.28 (0.18–0.72)	<0.001	1.51 (0.89–2.58)	0.13
Drug abuse or dependence	1.93 (1.37–2.72)	<0.001	0.45 (0.28–0.71)	0.001	0.96 (0.53–1.74)	0.88
Drug abuse or dependence^a^	3.00 (1.99–4.53)	<0.001	0.27 (0.15–0.48)	<0.001	1.22 (0.61–2.42)	0.57
Externalizing disorders	1.61 (1.25–2.06)	<0.001	0.35 (0.26–0.47)	<0.001	1.18 (0.81–1.73)	0.39
Externalizing disorders^a^	2.14 (1.56–2.94)	<0.001	0.29 (0.20–0.41)	<0.001	1.30 (0.82–2.07)	0.27

^a^adjusted for age, marital status, income, education, place of residence, immigrant
